# Decreased Functional Connectivity of the Core Pain Matrix in Herpes Zoster and Postherpetic Neuralgia Patients

**DOI:** 10.3390/brainsci13101357

**Published:** 2023-09-22

**Authors:** Jiaojiao Yang, Xiaofeng Jiang, Lili Gu, Jiahao Li, Ying Wu, Linghao Li, Jiaxin Xiong, Huiting Lv, Hongmei Kuang, Jian Jiang

**Affiliations:** 1 Department of Radiology, The First Affiliated Hospital of Nanchang University, 17 Yongwaizheng Street, Nanchang 330006, China; yangjjkkk@163.com (J.Y.); xiaofengjiang1245@163.com (X.J.); emailaddresswy@163.com (Y.W.); 13627090908@163.com (L.L.); xiong1301533133@163.com (J.X.); 17865599632@163.com (H.L.); kunghongmei@126.com (H.K.); 2 Neuroimaging Laboratory, Jiangxi Province Medical Imaging Research Institute, 17 Yongwaizheng Street, Nanchang 330006, China; 3 Department of Pain, The First Affiliated Hospital of Nanchang University, 17 Yongwaizheng Street, Nanchang 330006, China; lili.gutt@ncu.edu.cn; 4 Department of Neurology, The First Affiliated Hospital of Xi’an Jiaotong University, 277 Yanta West Road, Xi’an 710061, China; 029lee029@gmail.com

**Keywords:** neuropathic pain, pain matrix, fMRI, functional connectivity, machine learning

## Abstract

The purpose of this study was to explore the resting-state functional connectivity (FC) changes among the pain matrix and other brain regions in herpes zoster (HZ) and postherpetic neuralgia (PHN) patients. Fifty-four PHN patients, 52 HZ patients, and 54 healthy controls (HCs) underwent resting-state functional magnetic resonance imaging (rs-fMRI) scans. We used a seed-based FC approach to investigate whether HZ and PHN patients exhibited abnormal FC between the pain matrix and other brain regions compared to HCs. A random forest (RF) model was constructed to explore the feasibility of potential neuroimaging indicators to distinguish the two groups of patients. We found that PHN patients exhibited decreased FCs between the pain matrix and the putamen, superior temporal gyrus, middle frontal gyrus, middle cingulate gyrus, amygdala, precuneus, and supplementary motor area compared with HCs. Similar results were observed in HZ patients. The disease durations of PHN patients were negatively correlated with those aforementioned impaired FCs. The results of machine learning experiments showed that the RF model combined with FC features achieved a classification accuracy of 75%. Disrupted FC among the pain matrix and other regions in HZ and PHN patients may affect multiple dimensions of pain processing.

## 1. Introduction

Herpes zoster (HZ) is caused by the reactivation of varicella-zoster virus (VZV) latent in the dorsal root or cranial nerve ganglia and is characterized by a shingles rash on the skin corresponding to the affected nerve, often with severe pain at the site of the rash [1]. Postherpetic neuralgia (PHN) is the most common complication of HZ and is manifested by pain that lasts for one month or more after the shingle rash has healed [2]. Epidemiological surveys revealed that the incidence of PHN in HZ patients is usually 10~35%, and the higher incidence rates are associated with the elderly and the immune-impaired population [3]. Considering the long-term pain condition, PHN patients are prone to mental health problems such as anxiety, depression, and sleep disturbance, which seriously affect their quality of life [4]. PHN is a complex neuropathic pain disorder, the pathogenesis of which is still unclear, and there is a lack of reliable clinical measures to eradicate the pain [5].

In recent decades, with the rapid development of neuroimaging technology, rs-fMRI has been widely used to explore the central mechanisms associated with pain-related diseases as a rapid, noninvasive technique to study brain functional activity [6]. Studies show that pain is a complex and multifactorial subjective experience with extensive changes in cortical and subcortical structures during nociceptive processing, including the somatosensory cortex, thalamus, insula, anterior cingulate gyrus, frontal lobe, and parietal cortex. These brain areas are collectively known as the pain matrix [7,8]. Previous studies have shown that PHN is strongly associated with structural and functional abnormalities in the brain, which are located primarily in the pain matrix [9]. Cao et al. found increased brain activity in the thalamus and precentral gyrus in HZ and PHN patients [10]. In addition, they also found that PHN patients had lower local brain activity than HCs in bilateral inferior parietal lobules [11]. The insula is involved in the perception of injury and the cognitive and emotional aspects of pain regulation [12]. The study found that the gray matter volume (GMV) of the bilateral insula in HZ and PHN patients was lower than that in HCs and that the GMV of the left insula in PHN patients was negatively correlated with pain duration, suggesting that structural damage to the insula may lead to an extension of the duration of pain [13]. Maladaptive changes in the primary sensory cortex (S1) are characteristic of neuropathic pain [14]. Li et al. found that the functional connectivity (FC) of the bilateral upper S1 (SS1) in PHN patients was decreased, and the FC of the bilateral SS1 could predict the spontaneous pain intensity of patients [15]. The analysis of seed-based FC of the periaqueductal gray (PAG) showed that the FC between the PAG and anterior cingulate gyrus (ACC) was decreased in PHN patients, suggesting dysfunction of the descending pain regulation pathway in the brains of PHN patients [16]. Our recent study found that patients with HZ and PHN have significantly different network connectivities between the default mode, frontoparietal, cingulo-opercular, and sensorimotor networks [17].

Changes in the functional connection of the pain matrix during spontaneous pain in patients with HZ and PHN may be related to pain perception and processing, and the FC in the core pain matrix requires further study. Therefore, this study used pain matrix-related brain regions as core regions of pain processing. To evaluate the conversion mechanism of HZ and PHN, brain regions closely related to pain in PHN were selected as regions of interest (ROIs), including the bilateral thalamus, insula, anterior cingulate gyrus (ACC), inferior parietal lobule (IPL), precentral gyrus (primary motor cortex, M1), and postcentral gyrus (primary sensory cortex, S1). Furthermore, we systematically estimated differences in seed-based FC in the upper brain regions in patients with HZ and PHN compared to HCs and assessed the associations between altered brain connectivities and pain intensity, duration of disease, and emotion scores. In addition, the random forest (RF) machine learning algorithm was applied to explore the potential FC differences between the HZ and PHN. Therefore, we hypothesized that abnormal connection patterns may be present in the pain matrix and other regions. These findings might serve as neuroimaging markers for the transition from HZ to PHN patients.

## 2. Materials and Methods

### 2.1. Subjects

We used G*Power (version 3.1) and selected the F test to calculate the prior sample size of three groups at an alpha level of 0.05, a statistical power of 0.80, and an effect size of 0.43. The total number of samples needs to be at least 57 per group. All research procedures were reviewed and approved by the Medical Research Ethics Committee of the First Affiliated Hospital of Nanchang University and conducted in accordance with the ethical principles of the Helsinki Declaration. Subjects were recruited in the Department of Pain at the First Affiliated Hospital of Nanchang University. Before taking part in the study, all subjects obtained a complete explanation and completed written informed consent forms.

The clinical diagnoses of HZ and PHN were made by two consultant physicians from the Pain Department according to the standards of the International Association for Pain Research [18]. The participants recruited for this study included 55 PHN patients (61.8 ± 8.6 years), 55 HZ patients (61.0 ± 9.0 years), and 55 HCs (58.6 ± 6.3 years). The inclusion criteria for patients were as follows: (1) patients were right-handed; (2) HZ patients developed shingles within one month before admission, and the pain was not resolved; (3) PHN patients had pain that lasted for one month or more after the shingles rash healed; and (4) HZ or PHN patients had a pain score ≥ 5. The exclusion criteria for patients were as follows: (1) Herpes zoster in specific areas (eye, ear, or asymptomatic HZ); (2) suffering from other persistent acute or chronic pain; (3) suffering from neurological or psychiatric disorders; (4) having a history of head injury or alcohol or drug abuse; and (5) contraindications of MRI scanning. Fifty-five age- and sex-matched HCs were recruited, and HCs did not have any history of spontaneous pain or paresthesia, neurological or psychiatric illness, or drug abuse.

The clinical information of participants was collected before MRI scanning, and the patient’s pain intensity was assessed with the visual analog scale (VAS). The Hamilton Anxiety Scale (HAMA) and Hamilton Depression Scale (HAMD) were used to evaluate the anxiety and depression status of all participants.

### 2.2. Image Acquisition

MRI data were collected by a 3.0T Siemens Trio TIM Scanner (Erlangen, Bavaria, Germany) and an 8-channel phased array head orthogonal coil in the Radiology Department of the First Affiliated Hospital of Nanchang University. fMRI data were acquired using an echo-planar imaging sequence. There were the following parameters: repetition time (TR) = 2000 ms, echo time (TE) = 30 ms, flip angle = 90°, field of view (FOV) = 220 × 220 mm, matrix = 64 × 64, 30 interleaved axial slices, slice thickness = 4.0 mm, and scanned time points = 240. Three-dimensional high-resolution T1-weighted structural images with TR = 1900 ms, TE = 2.26 ms, flip angle = 9°, FOV = 215 × 230 mm, matrix = 240 × 256, 176 sagittal slices, and 1 mm slice thickness. During the rs-MRI scan, participants should close their eyes, try not to think, and avoid falling asleep.

### 2.3. Data Preprocessing

Preprocessing was performed using the Data Processing Assistant for rs-fMRI (DPARSF-V5.3, http://rfmri.org/DPARSF (accessed on 26 October 2022) and SPM12 (http://www.fl.ion.ucl.ac.uk/spm (accessed on 11 October 2022)) software based on MATLAB R2018a (The Math-Works, Natick, MA, USA). The following steps were included in the process: (1) DICOM format conversion; (2) removal of the first 10 time points to achieve magnetic field stabilization; (3) slice timing; (4) head motion correction (subjects whose head motions moved more than 2 mm in the X, Y, and Z axes or rotated more than 2° in each axis were excluded); and (5) segmentation and registration. The T1-weighted structural images and functional images of the participants were registered through linear transformation, and the transformed T1-weighted structural images were divided into white matter (WM), gray matter (GM), and cerebrospinal fluid (CSF) and normalized to the Montreal Neurological Institute (MNI) space. Then, the DARTEL [19] algorithm was used to normalize functional images to MNI space and resampled to 3 mm isotropic voxels; (6) spatial smoothing with a 6 mm FWHM; (7) filter (0.01~0.1 Hz) to reduce low-frequency drift and high-frequency physiological noise; (8) detrended linear drift; and (9) regression covariates (Friston-24 head motion parameters, WM, CSF, global signal) [20]. Finally, 1 HC, 1 PHN patient, and 3 HZ patients were excluded because of head motion greater than 2 mm or 2°. MR data from 54 PHN patients, 52 HZ patients, and 54 HCs continued for subsequent calculations.

### 2.4. Seed-Based Functional Connectivity Analysis

The thalamus, insula, ACC, IPL, M1, and S1 have been identified as important cortical areas for functional and structural alterations within the pain matrices of PHN patients [9,15]. The ROIs were selected from the Automated Anatomical Labeling (AAL) template, as the AAL template has been widely used in many neuroimaging studies [21]. Using the DPABI toolkit, we calculated Pearson correlation coefficients of the mean time series extracted from the AAL ROIs with other voxel signals from the whole brain to obtain a whole-brain FC map for each subject. The correlation maps were converted to Z value maps using the Fisher r-to-z transformation so that the data conform to a normal distribution.

### 2.5. Classifier and Performance Evaluation

Based on all the brain regions that differed among the three groups in the functional connectivity analysis described above, FC Z values from the corresponding brain regions were extracted and used to find potential FC differences that existed between HZ and PHN. In the scikit-learn-python library (https://scikit-learn.org/ (accessed on 16 December 2022)), a binary-based RF classifier model was created. By retaining the sample percentage of each class, the data set was randomly divided into 70% data for training and the remaining 30% for testing. The model parameters were optimized by using the grid search method. Evaluated classification performance on test sets with the best parameter models. To quantify the performance, the accuracy, sensitivity, and specificity of the model classification were calculated and evaluated using receiver operating characteristic (ROC) curves. To further evaluate the performance of the classifier, we performed a permutation test in which we randomized the class labels of the data before training and repeated the above process 10,000 times. A classifier is considered to perform well if the accuracy of the classifier trained with true class labels exceeds the 95% confidence interval of the accuracy of the classifier trained with random class labels [22]. Finally, we visualized feature importance, exploring possible FC differences between HZ and PHN by how much each pair of functional connectivity contributes to classification.

### 2.6. Statistical Analysis

SPSS 22.0 (SPSS Inc., Chicago, IL, USA) was used for statistical descriptive analysis of demographic and clinical data. The Shapiro–Wilk test was used to test the normality of all the data. Measurement data conforming to a normal distribution were expressed as the mean ± standard deviation; otherwise, the median (interquartile spacing) was used for statistical description. ANOVA was used for comparisons between the three groups, and the least significant difference method (LSD) *t* test was used for further between-group comparisons. The Mann–Whitney U test was used for comparisons between groups. Sex ratio and skin lesion comparisons were performed by the chi-square test. Statistical estimates were two-tailed, and a *p* value < 0.05 indicated that the differences were statistically significant.

Differences in FC z values between the three groups were assessed using one-way ANCOVA, and multiple comparisons were adjusted using Bonferroni’s method, with covariates including age and sex. Post hoc analyses were performed to further explore the differences in FC, with a two-tailed voxel-level *p* value of <0.001, and a cluster-level *p* value of <0.05. Gaussian random fields (GRF) were applied to correct thresholds for post hoc comparisons.

The FC z values with statistically significant differences were extracted for correlation analysis with the clinical data. Age and sex were used as covariates. Pearson correlation coefficients were calculated to determine the relationships among the continuous variables conforming to normality; otherwise, Spearman rank correlation coefficients were calculated, and the significance threshold was set as *p* < 0.05.

## 3. Results

### 3.1. Demographic and Clinical Features

There were no statistically significant differences in age (F = 2.337, *p* = 0.100) or sex (χ^2^ = 0.357, *p* = 0.837) among HZ, PHN patients, or HCs. There were no significant differences in patients’ VAS scores (Z = −0.695, *p* = 0.487) or skin lesions (χ^2^ = 0.336, *p* = 0.562). HAMA and HAMD scores were significantly higher in HZ and PHN patients than in HCs, and HAMD scores were higher in PHN patients than in HZ patients. Demographic and clinical data for all subjects are shown in Table 1.

### 3.2. Altered Functional Connectivity in Patients with HZ and PHN

**Figure 1:**
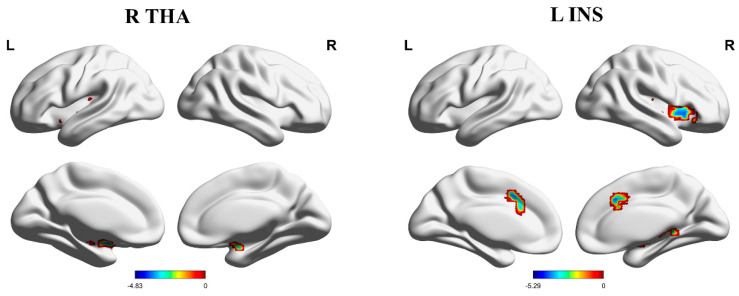
Brain areas indicating decreased functional connectivity between the right THA, left INS, and cerebral cortex in HZ patients compared to HCs. Notes: Gaussian random field correction, voxel-level *p* < 0.001, cluster-level *p* < 0.05, two-tailed. Abbreviations: L, left hemisphere; R, right hemisphere; THA, thalamus; INS, insula; HZ, herpes zoster; HCs, healthy controls.

**Figure 2:**
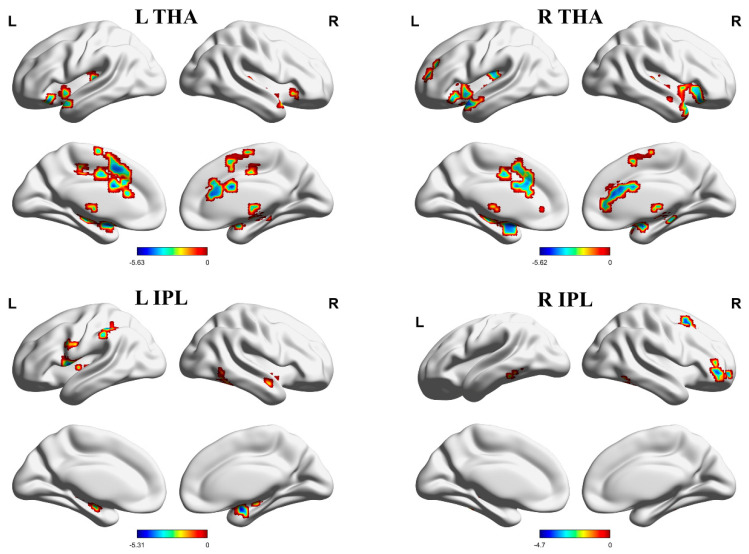
Brain areas indicating decreased functional connectivity between the THA, IPL, and cerebral cortex in PHN patients compared to HCs. Notes: Gaussian random field correction, voxel-level *p* < 0.001, cluster-level *p* < 0.05, two-tailed. Abbreviations: L, left hemisphere; R, right hemisphere; THA, thalamus; IPL, inferior parietal lobule; PHN, postherpetic neuralgia; HCs, healthy controls.

Both PHN and HZ patients’ functional connectivities were lower than those in HCs, but there were no significant differences between PHN and HZ patients. Specifically, compared with HCs, FCs between the right thalamus and bilateral putamen were reduced in HZ patients (Table 2, Figure 1), and FCs between the left thalamus and bilateral putamen, right inferior frontal gyrus, left superior temporal gyrus, and left ACC were decreased in PHN patients (Table 2, Figure 2). In addition, PHN patients had decreased FCs between the right thalamus and bilateral putamen, the left superior temporal gyrus, the left middle frontal gyrus, and the right ACC relative to HCs (Table 2, Figure 2).

**Figure 3:**
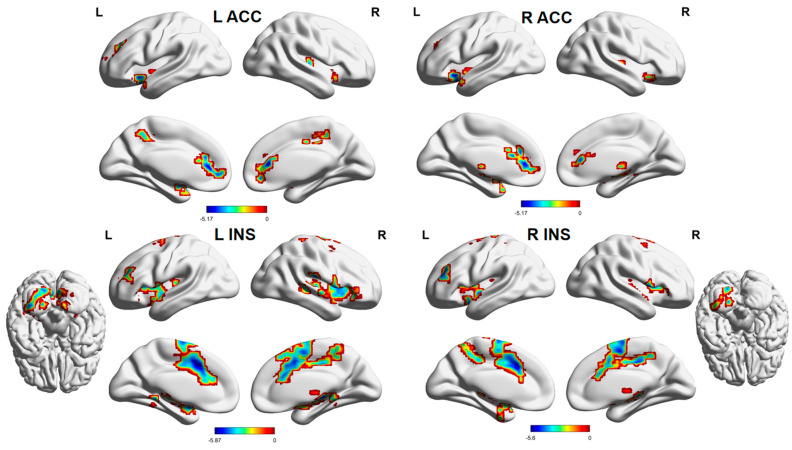
Brain areas indicating decreased functional connectivity between the ACC, INS, and cerebral cortex in PHN patients compared to HCs. Notes: Gaussian random field correction, voxel-level *p* < 0.001, cluster-level *p* < 0.05, two-tailed. Abbreviations: L, left hemisphere; R, right hemisphere; ACC, anterior cingulate gyrus; INS, insula; PHN, postherpetic neuralgia; HCs, healthy controls.

Compared with HCs, FCs between the left insula and right putamen and right middle cingulate gyrus were reduced in HZ patients (Table 2, Figure 1), and FCs between the left insula and the bilateral posterior cerebellum lobe, left middle frontal gyrus, and bilateral middle cingulate gyrus were decreased in PHN patients (Table 2, Figure 3). Moreover, PHN patients had decreased FCs between the right insula and the left posterior cerebellum lobe, the left insula, the left middle frontal gyrus, the left precuneus, and bilateral supplementary motor areas relative to HCs (Table 2, Figure 3).

Compared with HCs, FCs between the left ACC and the bilateral putamen and right medial cingulate gyrus, and FCs between the right ACC and the bilateral putamen were decreased in PHN patients (Table 2, Figure 3).

FCs between the left IPL and the right inferior temporal gyrus, right amygdala, and left M1, and FCs between the right IPL and the right inferior temporal gyrus, right middle frontal gyrus, and left fusiform were decreased in PHN patients relative to HCs (Table 2, Figure 2).

**Figure 4:**
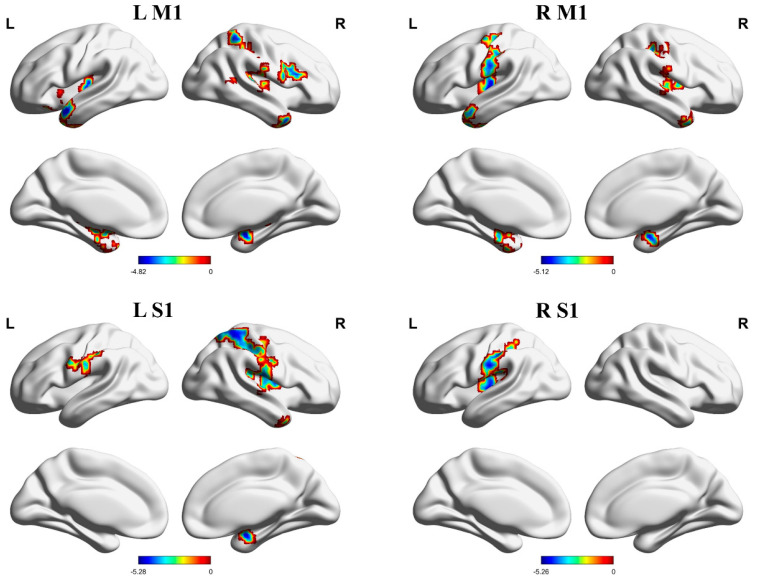
Brain areas indicating decreased functional connectivity between the M1, S1, and cerebral cortex in PHN patients compared to HCs. Notes: Gaussian random field correction, voxel-level *p* < 0.001, cluster-level *p* < 0.05, two-tailed. Abbreviations: L, left hemisphere; R, right hemisphere; M1, primary motor cortex; S1, primary sensory cortex; PHN, postherpetic neuralgia; HCs, healthy controls.

FCs between the left M1 and the right amygdala, left superior temporal gyrus, left insula, and right inferior frontal gyrus, and FCs between the right M1 and the left inferior temporal gyrus, left insula, right putamen, and bilateral S1 were decreased in PHN patients relative to HCs (Table 2, Figure 4).

Compared with HCs, FCs between the left S1 and the right amygdala and the right S1, and FCs between the right S1 and the left insula were decreased in PHN patients (Table 2, Figure 4).

### 3.3. Classification Results

The classification accuracy for discriminating PHN patients from HZ patients was 75% (*p* = 0.0035; sensitivity: 78.6%, specificity: 61.1%), AUC = 0.69 (Figure S1). Ranking the feature importance (Table S1), we found that the FC between the left M1 and the right inferior frontal gyrus and between the left thalamus and the left superior temporal gyrus contributed the most to the classification model.

### 3.4. Correlations with Clinical Features in HZ and PHN

**Figure 5:**
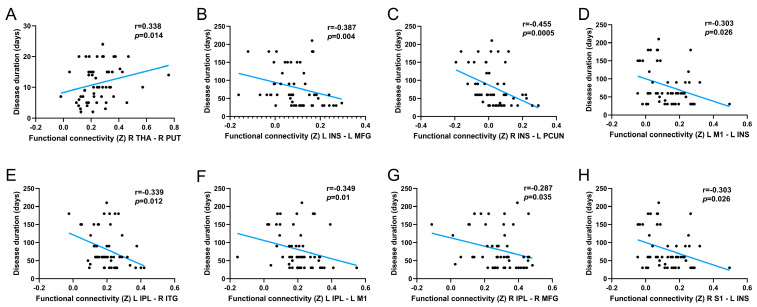
Scatter plot of the correlation between clinical variables and altered functional connectivity. (**A**) Correlation results for the HZ group. (**B**–**H**) Correlation results for the PHN group. Abbreviations: L, left hemisphere; R, right hemisphere; THA, thalamus; PUT, putamen; INS, insula; MFG, middle frontal gyrus; PCUN, precuneus; M1, primary motor cortex; IPL, inferior parietal lobule; ITG, inferior temporal gyrus; S1, primary sensory cortex.

The correlation analysis showed that the duration of disease in HZ patients was positively correlated with FC Z scores between the right thalamus and the right putamen (r = 0.338, *p* = 0.014, Figure 5A). In addition, negative correlations were found in the disease duration of the PHN patients and FC Z scores between the left insula and left middle frontal gyrus (r = −0.387, *p* = 0.004), right insula and left precuneus (r = −0.455, *p* = 0.0005), left M1 and left insula (r = −0.303, *p* = 0.026), left IPL and right inferior temporal gyrus (r = −0.339, *p* = 0.012), left IPL and left M1 (r = −0.349, *p* = 0.010), right IPL and right middle frontal gyrus (r = −0.287, *p* = 0.035), and right S1 and left insula (r = −0.303, *p* = 0.026) (Figure 5B–H).

## 4. Discussion

Previous studies have found that PHN patients had structural and functional abnormalities that were closely associated with brain regions located in the pain matrix (thalamus, insula, ACC, IPL, M1, S1). In this study, we systematically investigated the FCs between brain regions within the pain matrix and other brain regions. The results showed that compared with HCs, the FCs between those aforementioned regions were decreased in PHN patients. Similar results were observed in HZ patients. In addition, we applied the RF classifier to distinguish PHN from HZ and found that the model achieved an accuracy of 75%.

### 4.1. Altered Functional Connectivity with the Thalamus

The interaction of higher brain centers, including thalamocortical pathways, may be involved in the perception of pain and emotion [23]. Previous studies have found abnormal structural and functional connections in the thalamus of PHN patients, which may reflect impaired sensory monitoring in PHN patients [16]. Our study found that, compared with HCs, FCs between the thalamus and the bilateral putamen, left superior temporal gyrus, bilateral ACC, left middle frontal gyrus, and right inferior frontal gyrus were decreased in PHN patients. The putamen is helpful for the processing of pain sensations (such as intensity). Patients with putamen injury show decreased pain sensitivity and extensive activation of pain-related brain areas [24]. Functional and structural disorders affecting the putamen have been observed in PHN patients [25]. Decreased FC between the thalamus and the putamen may disrupt thalamocortical rhythms and lead to hyperalgesia in PHN patients. In addition, we found that the FC between the right thalamus and the bilateral putamen was decreased in HZ patients and that the decrease was related to the disease duration of the patients, thus potentially revealing the neural basis of pain sensory processing in patients in the acute phase. The ACC is associated with the aversive and unpleasant aspects of painful stimuli [26]. The thalamus transmits nociceptive signals through the ACC and participates in the emotional and motivational response to pain [27]. It was found that higher pain intensity and pressure pain sensitivity in patients with chronic musculoskeletal pain were associated with reduced gray matter in the superior temporal gyrus [28]. In our study, we found decreased FC between the thalamus and the left superior temporal gyrus in PHN patients, suggesting that the FC between those regions may be related to pain sensitivity and pain sensory processing. The middle frontal gyrus and inferior frontal gyrus, as part of the prefrontal cortex, are thought to be involved in the cognitive assessment and regulation of pain [29]. In addition, it was found that the left prefrontal lobe is involved in the sensory and emotional dimensions of pain experience [30]. Li et al. observed that pain intensity in PHN patients was associated with impaired FC in some subregions of the prefrontal lobe [31]. As evidence, our results show impaired FCs between the right thalamus and the left middle frontal gyrus and between the left thalamus and the right inferior frontal gyrus which may be associated with deficits in cognition and emotional regulation of pain in PHN patients.

### 4.2. Altered Functional Connectivity with the Insula and ACC

The insula and ACC, which are components of the limbic areas of the brain, play important roles in encoding pain-related emotions and motivation, and patients with abnormalities in these areas often show altered emotional responses to pain [27]. The insula is closely connected to a wide range of cortical and subcortical brain regions and is also involved in the processing of sensory, motivational, and cognitive functions [32]. The posterior cerebellum lobes are primarily associated with cognitive processing (e.g., learning) and emotional processing [33]. The middle cingulate gyrus is involved in pain, pain-induced negative effects, and related defensive behaviors [34]. The supplementary motor area is not only involved in motor planning and coordination of both sides of the body but is also involved in pain anticipation [35]. The precuneus is involved in different aspects of cognitive function, such as visuospatial imaging, situational memory retrieval, spontaneous thinking, and conscious processing, and it is associated with pain sensitivity [36]. The increased regional homogeneity (ReHo) and degree centrality (DC) in the left precuneus of PHN patients after short-term spinal cord stimulation (stSCS) may confirm the important role of the precuneus in pain relief [37]. In the present study, the FCs between the left insula and the right putamen and the right middle cingulate gyrus were significantly lower in HZ patients than in HCs, which may suggest processing in terms of emotional motivation in HZ patients. The PHN patients showed weaker FCs between the insula and the bilateral posterior cerebellum lobe, left middle frontal gyrus, bilateral middle cingulate gyrus, bilateral supplementary motor areas, and left precuneus, which may be related to the process of avoiding potential pain and harmful stimuli, as well as related cognitive and emotional evaluation and pain sensitivity. Interestingly, previous studies found a negative correlation between decreased insula GMV and disease duration in PHN patients [13]. As an additional measurement, we found that damaged FCs between the insula and multiple brain regions were also negatively correlated with disease duration. We speculate that the impairment of insula structure and function may be an important neuropathological basis for the development and maintenance of PHN.

We found that the FCs between the ACC and the putamen and middle cingulate gyrus were disrupted in PHN patients. The ACC is involved in pain regulation and the evaluation and expression of negative emotions. The putamen is a component of the striatum. Anatomically, the cingulate gyrus is connected to the striatum and is involved in the formation of cortical striatal thalamocortical circuits [38]. Our results may suggest pain-related emotional processing in patients with PHN.

### 4.3. Altered Functional Connectivity with IPL

In this study, we found that, compared to HCs, PHN patients had disrupted FCs between the IPL and the inferior temporal gyrus, amygdala, precentral gyrus, middle frontal gyrus, and fusiform gyrus. The IPL is involved in spatial discrimination and attention to pain [39], and its role in the neuropathology of PHN has been reported [11]. The inferior temporal gyrus is involved in higher cognitive functions, including visual and language comprehension and emotion regulation [40]. The fusiform gyrus is involved in higher visual processes [41]. The amygdala plays an important role in emotion processing [42]. In addition to controlling motor execution, the M1 is also associated with the ascending trigeminal-thalamocortical nociception pathway [43]. Disruption of FCs between the IPL and these brain regions may interfere with the regulatory process by which PHN patients intentionally shift their attention away from pain, leading to greater vulnerability to injurious stimuli. We also found that FC Z scores between the left IPL and the right inferior temporal gyrus and left M1 and between the right IPL and the right middle frontal gyrus were negatively correlated with disease duration. This suggests that disease progression in PHN patients may drive functional changes, resulting in disruption of the connectivity between the IPL and the frontal lobe.

### 4.4. Altered Functional Connectivity with M1 and S1

We found that the M1 and S1 in PHN patients had decreased FCs in many other brain regions, including the right amygdala, left insula, left superior temporal gyrus, left inferior temporal gyrus, right inferior frontal gyrus, and right putamen. Interestingly, all of these brain regions are mentioned in the above neuropathology involving PHN patients. In addition, we also found FC disruptions within the sensorimotor cortex, where the FC of the bilateral S1 was lower in PHN patients than in HCs, which is consistent with previous studies [15]. FC of the sensorimotor cortex correlates with pain sensitivity [44]. It is speculated that these impaired FCs may imply impaired control of movement or be related to competition between neural networks (i.e., neural networks related to sensorimotor function are losing influence, while those related to pain and protection are gaining influence) [45].

### 4.5. Identifying Potential Functional Connectivity Differences between PHN and HZ Patients

We found no significant difference in FC between the two groups of patients, possibly due to the relatively short disease duration of the included PHN patients or the stringent correction method for GRF (voxel level: *p* < 0.001, cluster level: *p* < 0.05). In recent years, machine learning combined with diverse neuroimaging features to identify previously unknown key factors or interactions has been increasingly applied to study different types of pain disorders [46,47]. RF is a widely used machine learning method, and the algorithm also provides the corresponding weights for each variable to reveal the contribution of these variables to the model. Using the RF classifier, we found that the FC between the left M1 and the right inferior frontal gyrus and the left thalamus and the left superior temporal gyrus had the ability to potentially distinguish PHN patients from HZ patients, with 75% accuracy of classification (*p* = 0.0035; sensitivity: 78.6%, specificity: 61.1%). Previous studies have found increased brain activity in the M1 and inferior frontal gyrus, increased thalamic GMV, and decreased temporal lobe GMV in PHN patients when HZ progresses to PHN, suggesting that functional and structural changes in these brain regions may be correlated with HZ-PHN chronification [48]. Our findings provide further support for the possibility that changes in these brain areas may be the cause of refractory neuralgia in patients with PHN.

### 4.6. Limitations

There are some limitations to this study. First, this is a cross-sectional study, and our results need to be further validated and refined by longitudinal studies. Second, our small sample size influenced the analysis of changes in other neuroimaging measurements. Third, we assessed pain matrix internetwork changes, and further studies are needed to investigate pain matrix intranetwork changes. Fourth, we did not delve into pain transmission pathways in PHN patients. Fifth, there are few investigations on brain network graph theory in HZ and PHN patients. Sixth, this study lacks clinical evidence to support potential abnormalities in some network functions. Seventh, we did not collect PHN patients with paresthesias for analysis. In the future, we will make more scientific contributions in these aspects.

## 5. Conclusions

HZ and PHN patients had disrupted FCs among the pain matrix and putamen, superior temporal gyrus, middle frontal gyrus, middle cingulate gyrus, amygdala, precuneus, and supplementary motor area. In addition, those aforementioned abnormal FCs were correlated with disease duration. The RF classifier was 75% accurate in distinguishing between HZ and PHN patients. The feature of functional connectivity between the left primary motor cortex and the right inferior frontal gyrus had the greatest weight. These findings suggest that the pain matrix plays a key role in the complex pathophysiology of HZ and PHN patients.

## Figures and Tables

**Table 1:** Demographic data and clinical features.

Clinical Information	HC	HZ	PHN	Statistic Results (*p* Values)
	*n* = 54	*n* = 52	*n* = 54	
Age (years)	58.6 ± 6.3	61.0 ± 9.0	61.8 ± 8.6	F = 2.337 (0.100)
Sex (male: female)	27:27	24:28	28:26	χ^2^ = 0.357 (0.837)
Skin lesions (L: R)	—	27:25	25:29	χ^2^ = 0.336 (0.562)
Illness duration (days)	—	10.0 (8.8)	60.0 (90.0) ^a^	Z = −8.908 (<0.001) *
VAS score	—	6.0 (1.0)	6.0 (1.0)	Z = −0.695 (0.487)
HAMA score	6.9 ± 1.8	12.9 ± 3.6 ^b^	13.0 ± 3.3 ^c^	F = 74.692 (<0.001) *
HAMD score	6.6 ± 1.9	15.4 ± 3.8 ^b^	17.9 ± 5.4 ^a,c^	F = 119.813 (<0.001) *

Notes: Data satisfying a normal distribution are described as the mean ± standard deviation; otherwise, they are expressed as the median (interquartile spacing). F, Z, and χ^2^ denote the ANOVA, Mann–Whitney U test, and chi-square test statistics, respectively. * Significant difference among PHN, HZ, and HC; ^a^ significant difference between PHN and HZ; ^b^ significant difference between HZ and HC; ^c^ significant difference between PHN and HC. Abbreviations: HC, healthy control; HZ, herpes zoster; PHN, postherpetic neuralgia; VAS, visual analog scale; HAMA, Hamilton Anxiety Scale; HAMD, Hamilton Depression Scale.

**Table 2:** Brain regions with decreased functional connectivity (FC) in HZ and PHN patients compared to HCs.

Regions of Interest	Contrast	Brain Region		Cluster Size (Number of Voxels)	Peak Intensity	MNI Coordinates		
						X	Y	Z
Left THA	PHN < HC	Inferior Frontal Gyrus	R	102	−5.10	54	21	−9
		Superior Temporal Gyrus	L	128	−5.32	−45	18	−18
		Putamen	L	629	−5.21	−12	9	−3
		Putamen	R	503	−5.63	15	0	9
		ACC	L	438	−4.91	0	9	24
Right THA	HZ < HC	Putamen	R	66	−4.33	21	18	0
		Putamen	L	131	−4.83	−36	−12	−6
	PHN < HC	Putamen	R	863	−5.62	12	6	0
		Putamen	L	761	−4.96	−27	−15	9
		Superior Temporal Gyrus	L	142	−5.15	−45	18	−18
		ACC	R	383	−4.71	6	24	21
		Middle Frontal Gyrus	L	84	−4.78	−33	51	15
Left INS	HZ < HC	Putamen	R	362	−5.29	30	9	9
		Median Cingulate Gyrus	R	128	−4.34	3	21	33
	PHN < HC	Bilateral Cerebellum Posterior Lobe		1057	−5.46	−36	−57	−57
		Middle Frontal Gyrus	L	111	−4.63	−33	45	21
		Bilateral Median Cingulate Gyrus		1324	−5.87	−9	12	39
Right INS	PHN < HC	Cerebellum Posterior Lobe	L	295	−5.60	−36	−57	−57
		Insula	L	663	−5.52	−42	−3	−6
		Middle Frontal Gyrus	L	128	−4.74	−33	42	18
		Bilateral Supplementary Motor Area		996	−5.16	−12	−9	54
		Precuneus	L	113	−4.24	−12	−45	54
Left ACC	PHN < HC	Putamen	L	340	−5.16	−24	−12	0
		Putamen	R	260	−4.64	18	18	−6
		Median Cingulate Gyrus	R	141	−4.47	6	−33	45
Right ACC	PHN < HC	Putamen	L	408	−5.17	−24	−12	0
		Putamen	R	290	−4.55	18	18	−6
Left IPL	PHN < HC	Inferior Temporal Gyrus	R	134	−5.30	51	−54	−15
		Amygdala	R	124	−4.75	27	0	−18
		M1	L	102	−4.53	−51	3	9
Right IPL	PHN < HC	Inferior Temporal Gyrus	R	104	−4.70	51	−54	−15
		Fusiform Gyrus	L	90	−4.69	−45	−57	−21
		Middle Frontal Gyrus	R	75	−4.30	33	9	51
Left M1	PHN < HC	Amygdala	R	154	−4.82	30	0	−21
		Superior Temporal Gyrus	L	152	−4.69	−51	6	−18
		Insula	L	301	−4.64	−33	−18	3
		Inferior Frontal Gyrus	R	126	−4.61	51	21	18
Right M1	PHN < HC	Inferior Temporal Gyrus	L	172	−4.60	−42	9	−36
		Insula	L	135	−5.12	−39	−15	3
		Putamen	R	120	−4.22	30	−12	12
		S1	L	209	−4.56	−51	−18	33
		S1	R	120	−4.33	42	−30	36
Left S1	PHN < HC	Amygdala	R	85	−4.89	27	−3	−18
		S1	R	690	−5.28	36	−42	60
Right S1	PHN < HC	Insula	L	359	−5.25	−39	−15	3

Notes: Gaussian random field correction, voxel-level *p* < 0.001, cluster-level *p* < 0.05, two-tailed. Abbreviations: L, left hemisphere; R, right hemisphere; THA, thalamus; INS, insula; ACC, anterior cingulate gyrus; IPL, inferior parietal lobule; M1, primary motor cortex; S1, primary sensory cortex.

## Data Availability

Data related to this study may be provided upon reasonable request by the corresponding authors.

## Supplementary Materials

The following supporting information can be downloaded at: https://www.mdpi.com/article/10.3390/brainsci13101357/s1, Figure S1: Receiver operating characteristic (ROC) curve of the random forest (RF) classifier used to distinguish patients with HZ and PHN; Table S1: Functional connectivity feature importance in the random forest (RF) algorithm used to distinguish HZ and PHN patients.
