# Supplementary material for: Decreased Functional Connectivity of the Core Pain Matrix in Herpes Zoster and Postherpetic Neuralgia Patients
Source: Brain Sci. 2023 Sep 22;13(10):1357. doi: 10.3390/brainsci13101357 (PMC10605464; doi:10.3390/brainsci13101357)
Supplement: Supplementary file 1 [file brainsci-13-01357-s001.zip › brainsci-2557391-supplementary.pdf]

supplementary materials

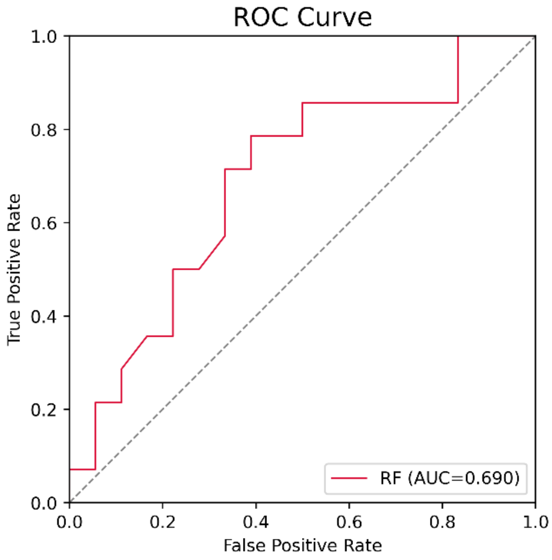

Figure S1. Receiver operating characteristic (ROC) curve of the random forest (RF) classifier used to distinguish patients with HZ and PHN.

Table S1. Functional connectivity feature importance in the random forest (RF) algorithm used to distinguish HZ and PHN patients

**Brain region**	**Feature importance**
M1.L - IFG.R	0.156598
THA.L - STG.L	0.128122
ACC.L - DCG.L	0.081678
INS.L - CPL	0.079729
M1.R - INS.L	0.073820
THA.L - PUT.R	0.056698
ACC.R - PUT.R	0.050660
M1.L - INS.L	0.048118
INS.R - PCUN.L	0.043700
M1.R – S1.L	0.042467
M1.R – S1.R	0.039940
IPL.L - ITG.R	0.036231
THA.R - ACC.R	0.030006
INS.L - DCG	0.021296
INS.L - MFG.L	0.021235
THA.R - PUT.L	0.020425
ACC.L - PUT.R	0.018853
M1.R - PUT.R	0.014586
THA.L - ACC.L	0.013286
INS.R - MFG.L	0.009370
INS.R - INS.L	0.006131
M1.R - ITG.L	0.005354
INS.R - SMA	0.001697

Abbreviations: R, right hemisphere; L, left hemisphere; M1, primary motor cortex; IFG, inferior frontal gyrus; THA, thalamus; STG, superior temporal gyrus; ACC, anterior cingulate gyrus; DCG, middle cingulate gyrus; INS, insula; CPL, posterior cerebellum lobe; PUT, putamen; PCUN, precuneus; S1, primary sensory cortex; IPL, inferior parietal lobule; ITG, inferior temporal gyrus; MFG, middle frontal gyrus; SMA, supplementary motor area.
